# Electrocardiogram derived heart age models agreement, accuracy and predictive ability in the Tromsø study

**DOI:** 10.1038/s41514-026-00344-2

**Published:** 2026-03-20

**Authors:** Arya Panthalanickal Vijayakumar, Tom Wilsgaard, Henrik Schirmer, Ernest Diez Benavente, René van Es, Rutger R. van de Leur, Haakon Lindekleiv, Zachi I. Attia, Francisco Lopez-Jimenez, David A. Leon, Olena Iakunchykova

**Affiliations:** 1 Department of Community Medicine, UiT The Arctic University of Norway, Tromsø, Norway; 2 Akershus University Hospital, Lørenskog, Norway; 3 Institute of Clinical Medicine, Campus Ahus, University of Oslo, Oslo, Norway; 4 Department of Experimental Cardiology University Medical Center Utrecht, Utrecht, The Netherlands; 5 Department of Cardiology University Medical Center Utrecht, Utrecht, The Netherlands; 6 Department of Radiology, University Hospital of North, Oslo, Norway; 7 Mayo Clinic College of Medicine, Rochester, MN USA; 8 Department of Noncommunicable Disease Epidemiology, London School of Hygiene & Tropical Medicine, London, United Kingdom; 9 Department of Psychology, University of Oslo, Oslo, Norway

**Keywords:** Cardiology, Diseases, Medical research, Risk factors

## Abstract

Convolutional neural networks (CNNs) can estimate electrocardiogram (ECG)-based heart age. We compared three published CNNs in the Tromsø Study cohort (7,108 participants) for accuracy, agreement, and prognostic value. Mean absolute error versus chronological age was 6.8, 7.8, and 6.4 years. Correlations with age were ~0.71–0.73 and agreement across CNNs was high (overall ICC 0.86). Using Cox models, we estimated hazard ratios per SD of δ-age (ECG age minus chronological age) for myocardial infarction, stroke, cardiovascular mortality, and all-cause mortality; discrimination was quantified by cross-validated C-index. δ-age predicted higher risk across outcomes; associations were strongest for δ-age_1_ with myocardial infarction and all-cause mortality (HR 1.36 (1.11, 1.67) and 1.27 (1.08, 1.50)) and for δ-age_2_ with stroke and cardiovascular mortality (HR 1.45 (1.17, 1.80) and 1.48 (1.07, 2.05)). C-indices were similar across models. Despite architectural and training-set differences, CNNs yielded consistent ECG ages and comparable risk prediction in an external population.

## Introduction

Biological aging encapsulates the physiological and molecular changes an organism undergoes over its lifespan. The quantification of biological age, as opposed to chronological age, offers potentially profound insights into an individual’s health status and predisposition to age-related diseases^1,2^. Various biomarkers of biological age have been developed, including telomere length, the epigenetic clock, mitochondrial DNA copy number, inflammatory clock, and biomarkers specific to certain organs and systems^3–7^. These biomarkers provide a multidimensional view of aging, reflecting the complex interplay of genetic, environmental, and lifestyle factors that influence the aging process. They may help to unravel the complex mechanisms of aging and assist with developing targeted interventions. In clinical settings, these biological age estimates can inform personalized prognosis, improve early detection of age-related diseases and tailor treatments to individual patient^8^.

The methodologies for estimating the biological age have evolved dramatically with the advent of artificial intelligence. Earlier biomarkers of aging, that relied on clinical and biochemical parameters, have provided foundational insights but often lacked the precision and organ specificity afforded by modern deep learning (DL)^9^. This shift in approach is mirrored in cardiovascular medicine, where convolutional neural networks (CNNs) applied to raw electrocardiograms (ECG) allow reproducible estimation of biological age of the heart called ECG age^10^. Unlike traditional methods that might rely on a composite of risk factors and clinical assessments^11^, CNN captures the entire information provided by ECG waveforms, translating them into a metric of biological age^3,12^. This metric can be used to calculate the δ-age, which represents the deviation from chronological age for each individual. A positive δ-age indicates that a heart is biologically older than what would be expected from its chronological age. Importantly, δ-age has been shown to predict risk of adverse health outcomes and mortality^3,12^.

With CNN gaining popularity, several models of ECG age have been proposed^3,13–15^. They differ depending on the model architecture used, model parameters specified (like network depth, loss function), population they have been trained and validated on and characteristics of the ECG recordings. However, there have been no previous attempts to examine how far different CNNs applied to the same set of ECGs will result in different values of the ECG age.

The aim of this study is to compare ECG age outputs from three CNNs that are different by architecture, population where training and testing data originate from, or both. We use data from 7108 participants of the Tromsø Study who were followed up for cardiovascular outcomes over a period of 6 years. To compare these ECG ages, we looked at their correlation and accuracy in the prediction of the chronological ages, agreement between each other, and agreement in the ability to predict cardiovascular outcomes and mortality. Such a comparison is needed to guide for future research and implementation of biomarker of heart aging in clinical studies, for example, to predict the risk of adverse health outcomes or to test the effect of preventive interventions.

## Results

**Fig. 1:**
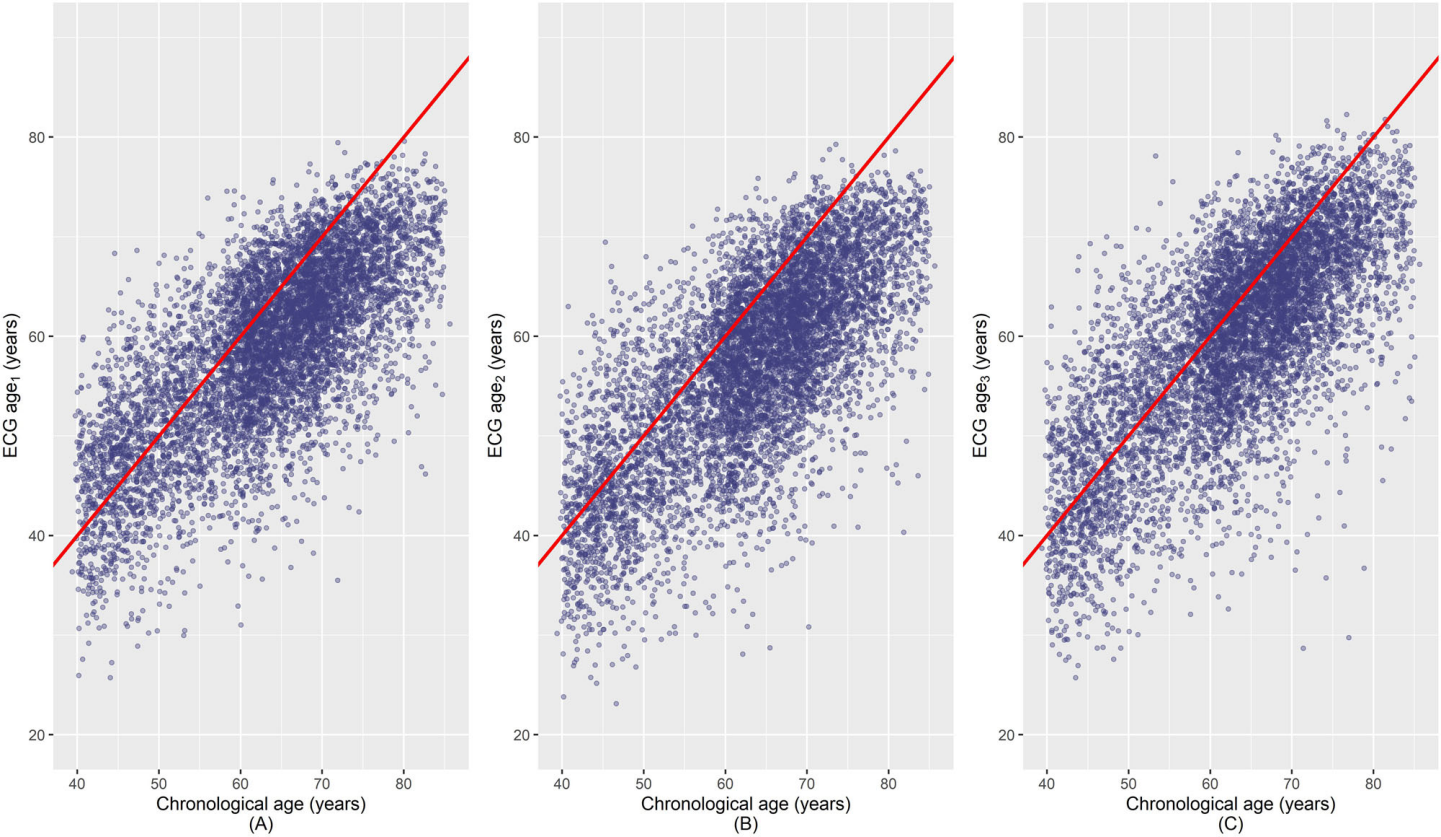
The scatterplot between chronological age and ECG age estimated by three convolutional neural networks (CNNs). **A** ECG age_1_: estimated by CNN1 model trained, tested and validated with data from Mayo Clinic, MN, USA. **B** ECG age_2_: estimated by CNN2 model based on Mayo clinic’s model architecture and trained, tested and validated with data from Utrecht UMC, Netherlands. **C** ECG age_3_: estimated by CNN3 model based on CausalCNN architecture and trained, tested and validated with data from Utrecht UMC, Netherlands.

For the sample of 7108 individuals the mean chronological age was 63.2 (SD 10.4), the mean ECG age_1_ was 58.7 years (SD 8.9), ECG age_2_ was 57.0 (SD 9.6) and ECG age_3_ was 59.7 (SD 9.9) (Table 1, Fig. 1). The corresponding means (SDs) for the δ-ages are 4.4 (7.3), 6.1 (7.6) and 3.4 (7.5), respectively.

**Table 1:** Descriptive characteristics of the study sample: the Tromsø study 2015–2021

Characteristic	Mean (SD) or *N* (%)
ECG age_1_^a^ (years), mean (SD)	58.7 (8.9)
ECG age_2_^a^ (years), mean (SD)	57.0 (9.6)
ECG age_3_^a^ (years), mean (SD)	59.7 (9.9)
δ-age_1_^b^ (years), mean (SD)	-4.4 (7.3)
δ-age_2_^b^ (years), mean (SD)	-6.1 (7.6)
δ-age_3_^b^ (years), mean (SD)	-3.4 (7.5)
Baseline covariates	
Age (years), mean (SD)	63.2 (10.4)
Sex (female), *N* (%)	4019 (56.5%)
Systolic BP (mmHg), mean (SD)	133.3 (20.2)
Non-HDL cholesterol (mmol/L), mean (SD)	3.94 (1.07)
Daily smokers, %	892 (12.5%)

^a^ECG age_1_: estimated by CNN1 model, trained, tested and validated with data from Mayo Clinic, MN, USA; ECG age_2_: estimated by CNN2 model, trained, tested and validated with data from Utrecht UMC, Netherlands; ECG age_3_: estimated by CNN3 model based on CausalCNN architecture and trained, tested and validated with data from Utrecht UMC, Netherlands.

^b^δ-age_1_ calculated as the difference between ECGage_1_ and chronological age; δ-age_2_ calculated as the difference between ECGage_2_ and chronological age; δ-age_3_ calculated as the difference between ECGage_3_ and chronological age.

The baseline covariates in Table 1 include systolic blood pressure (mean 133.3 mmHg), non-HDL cholesterol (mean 3.94 mmol/l), and daily smoking (12.5% of the participants).

### Accuracy of CNN models for ECG age

The mean absolute error (MAE) between the ECG age and the chronological age was 6.82 for ECG age_1_, which was similar to that obtained for the CNN in the original publication^3^ on the holdout dataset (6.9 years), 7.82 for ECG age_2_ (7.2 years at initial validation during model development), and 6.41 for ECG age_3_ (7.0 years at initial validation during model development).

### Agreement between CNN models for ECG age

**Fig. 2:**
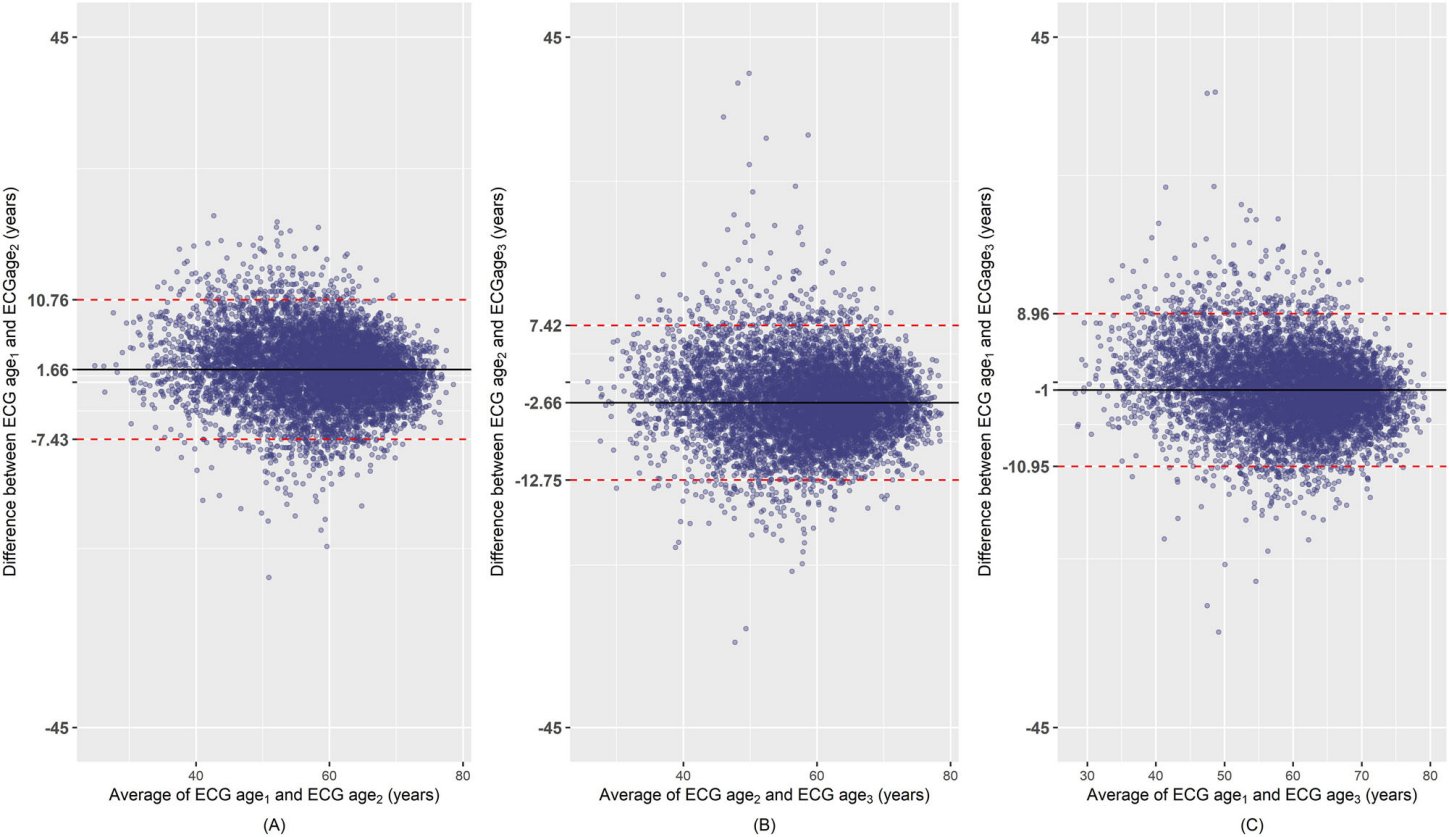
The Bland–Altman plots demonstrating the pairwise agreement between ECG ages with 95% limits of agreement. **A** ECG age_1_ v/s ECG age_2_, **B** ECG age_2_ v/s ECG age_3_, and **C** ECG age_1_ v/s ECG age_3_. ECG age_1_ estimated by CNN1 model with data from Mayo Clinic, MN, USA, ECG age_2_: estimated by CNN2 model from Mayo Clinic, MN, USA, with data from Utrecht UMC, Netherlands; ECG age_3_: estimated by CNN3 model with data from Utrecht UMC, Netherlands.

BA plot demonstrated a fair agreement between ECG age estimations done with different CNN models (Fig. 2). The visual inspection of the histograms of pairwise differences and the BA plots indicated that the Bland–Altman assumptions were satisfied: the differences were approximately normally distributed and the variability was consistent across the range of values. The absolute values of mean difference between the ECG ages ranged between 1.0 and 2.7 for each comparison indicating a slight bias and the limits of agreement ranged from approximately 9 years above and below the mean difference. Figure 2 also shows that most data points are clustered closely around the mean difference, indicating a fair agreement. Pearson’s correlation coefficient between ECG age and chronological age was 0.72, 0.71, and 0.73 for the ECG age_1_, ECG age_2_ and ECG age_3_, respectively. Additionally, the correlation coefficients for the pairs (ECG age_1_, ECG age_2_), (ECG age_2_, ECG age_3_), and (ECG age_1_, ECG age_3_) were 0.88, 0.86 and 0.86, respectively (Table 2, Supplementary Fig. 1). The ICC as a numeric estimate of agreement for the same pairs of ECG ages were 0.87 (95% CI: 0.87, 0.88), 0.86 (95% CI: 0.85, 0.87) and 0.85 (95% CI: 0.85, 0.86), respectively, with an overall ICC of 0.86 (95% CI: 0.86, 0.87). The coefficient of variation, CV, for the same pairs was 6.0% (95% CI: 5.9, 6.1), 7.0% (95% CI: 6.9, 7.1), and 6.2% (95% CI: 6.1, 6.3), respectively with an overall is 6.4% (95% CI: 6.4, 6.5). The Lin’s concordance correlation coefficients (CCCs) and calibration slopes for each model pair are shown in Supplementary Table 1. The CCCs were very high (around 0.83–0.86) and the calibration slopes were around 0.77–0.96 for all comparisons, indicating strong agreement and minimal scaling bias between models.

**Table 2:** Pearson’s (r) and intra-class correlation coefficients (ICC) and coefficient of variation (CV) between ECG ages

	*r*	CV% (95% CI)	ICC (95% CI)
ECG age_1_ vs ECG age_2_	0.88	6.02 (5.92, 6.12)	0.87 (0.87, 0.88)
ECG age_2_ vs ECG age_3_	0.86	7.02 (6.90, 7.13)	0.86 (0.85, 0.87)
ECG age_1_ vs ECG age_3_	0.86	6.18 (6.08, 6.28)	0.85 (0.85, 0.86)
Overall		6.42 (6.35, 6.49)	0.86 (0.86-0.87)

The Tromsø Study 2015–16.

### Prediction of CVD morbidity and mortality using δ-age

Among 7108 Tromsø7 participants, we observed 156 (2.2%) incident fatal and non-fatal MIs, 141 (1.9%) incident fatal and non-fatal strokes, 66 (0.9%) CVD deaths, and 251 (3.5%) total deaths during a 6-year median follow-up time.

Table 3 shows hazard ratios (HRs) for the association between δ-age and outcomes. For MI δ-age_1_ had the strongest multivariable adjusted HR, 1.36 (95% CI: 1.11, 1.67) while δ-age_3_ had the weakest, 1.17 (0.97, 1.42). In other words, each extra SD of δ-age_1_ was associated with a 36% increased risk of MI, and each extra SD of δ-age_3_ was associated with a 17% increased risk of MI. For stroke, the strongest HR was observed for δ-age_2_,1.45 (1.17, 1.80) and the weakest for δ-age_1_,1.33 (1.07, 1.67). For CVD mortality, the strongest HR was observed for δ-age_2_, 1.48 (1.07, 2.05) and the weakest for δ-age_3_, 1.09 (0.81, 1.48). For total mortality, δ-age_1_ had the strongest HR, 1.27 (1.08, 1.50), while δ-age_2_ had the weakest, 1.21 (1.04, 1.41). HRs adjusted for only age and sex were almost identical to HRs further adjusted for systolic BP, non-HDL cholesterol and daily smoking.

**Table 3:** Hazard ratios with 95% confidence intervals for δ-ages calculated from different CNNs

	δ-age_1_^a^		δ-age_2_^a^		δ-age_3_^a^	
*N* = 7108	Age and sex adjusted	Multivariable adjusted^b^	Age and sex adjusted	Multivariable adjusted^b^	Age and sex adjusted	Multivariable adjusted^b^
MI	1.39 (1.13, 1.70)	1.36 (1.11, 1.67)	1.23 (1.02, 1.48)	1.20 (1.00, 1.46)	1.20 (0.99, 1.46)	1.17 (0.97, 1.42)
Stroke	1.36 (1.09, 1.69)	1.33 (1.07, 1.67)	1.48 (1.19, 1.83)	1.45 (1.17, 1.80)	1.46 (1.18, 1.81)	1.42 (1.14, 1.75)
CVD mortality	1.40 (1.00, 1.95)	1.40 (1.00, 1.95)	1.48 (1.07, 2.05)	1.48 (1.07, 2.05)	1.09 (0.81, 1.47)	1.09 (0.81, 1.48)
Total mortality	1.27 (1.08, 1.49)	1.27 (1.08, 1.50)	1.20 (1.03, 1.40)	1.21 (1.04, 1.41)	1.20 (1.03, 1.40)	1.22 (1.05, 1.43)

The Tromsø study 2015–2021.

^a^δ-age_1_ calculated as the difference between ECGage_1_ and chronological age, per standard deviation; δ-age_2_ calculated as the difference between ECGage_2_ and chronological age, per standard deviation; δ-age_3_ calculated as the difference between ECGage_3_ and chronological age, per standard deviation; ECG age_1_: estimated by CNN1 model trained, tested and validated with data from Mayo Clinic, MN, USA, ECG age_2_: estimated by CNN2 model trained, tested and validated with data from Utrecht UMC, Netherlands; ECG age_3_: estimated by CNN3 model based on CausalCNN architecture and trained, tested and validated with data from Utrecht UMC, Netherlands.

^b^In addition to age and sex, this model is adjusted for daily smoking, systolic BP and non-HDL cholesterol.

The BA plots in Supplementary Figs. 2–5 demonstrated fair agreements between the predicted 6-year survival probabilities from the aforementioned models for all four outcomes. For total and CVD mortality, the BA plot had a wider level of agreement as compared to that of MI and stroke for all the pairwise comparisons. The ICC of the same 6-year survival probabilities were 0.98 (0.97, 0.98) for MI, 0.96 (0.96, 0.96) for stroke, 0.97 (0.97, 0.97) for CVD mortality and 0.99 (0.99, 0.99) for total mortality. Table 4 shows the C index for each outcome from separate Cox models that include chronological age and one of the following ECG-age specifications: ECG age_1_, ECG age_2_, ECG age_3_, the average of the three, or all three as separate predictors in a single model. The indices varied somewhat between the outcomes, but all demonstrated good discriminatory power. C indices did not differ significantly between the any pair of the models.

**Table 4:** C-index calculated by 8-fold cross validation on the Cox model, with age and different ECG age specifications as predictors of myocardial infarction, stroke, cardiovascular mortality and total mortality

ECG-age specification	MI	Stroke	CVD mortality	Total mortality
C index				
ECG age_1_^a^	0.670	0.730	0.819	0.756
ECG age_2_^a^	0.663	0.735	0.819	0.753
ECG age_3_^a^	0.663	0.731	0.815	0.753
Mean ECG age^b^	0.667	0.734	0.818	0.755
All 3 ECG ages^c^	0.668	0.733	0.818	0.754
Pairwise difference in C index (95% CI)				
ECG age_1_ vs. ECG age_2_	0.007 (−0.004, 0.020)	−0.005 (−0.017, 0.005)	0.000 (−0.012, 0.011)	0.002 (−0.001, 0.007)
ECG age_2_ vs. ECG age_3_	0.000 (−0.010, 0.010)	0.004 (−0.009, 0.018)	0.004 (−0.008, 0.019)	0.000 (−0.004, 0.005)
ECG age_1_ vs. ECG age_3_	−0.007 (−0.022, 0.004)	0.002 (−0.008, 0.013)	−0.003 (−0.014, 0.005)	−0.003 (−0.009, 0.002)
Mean ECG age vs. ECG age_1_	−0.003 (−0.012, 0.005)	0.004 (−0.002, 0.013)	−0.001 (−0.007, 0.004)	−0.001 (−0.004, 0.002)
Mean ECG age vs. ECG age_2_	0.004 (−0.002, 0.012)	−0.001 (−0.009, 0.007)	−0.001 (−0.011, 0.006)	0.001 (−0.001, 0.005)
Mean ECG age vs. ECG age_3_	0.004 (−0.002, 0.013)	0.003 (−0.005, 0.012)	0.002 (−0.004, 0.011)	0.001 (−0.001, 0.006)
All 3 ECG ages vs. ECG age_1_	−0.002 (−0.008, 0.007)	0.003 (−0.007, 0.017)	−0.001 (−0.015, 0.017)	−0.002 (−0.005, 0.002)
All 3 ECG ages vs. ECG age_2_	0.005 (−0.007, 0.021)	−0.002 (−0.009, 0.008)	−0.001 (−0.013, 0.013)	0.001 (−0.003, 0.007)
All 3 ECG ages vs. ECG age_3_	0.005 (−0.007, 0.021)	0.001 (−0.006, 0.015)	0.003 (−0.015, 0.022)	0.001 (−0.003, 0.007)
All 3 ECG ages vs. Mean ECG age	0.001 (−0.008, 0.015)	−0.001 (−0.008, 0.008)	0.000 (−0.016, 0.019)	−0.001 (−0.004, 0.004)

The Tromsø study 2015–2021.

^a^ECG age_1_: estimated by CNN1 model trained, tested and validated with data from Mayo Clinic, MN, USA, ECG age_2_: estimated by CNN2 model trained, tested and validated with data from Utrecht UMC, Netherlands; ECG age_3_: estimated by CNN3 model based on CausalCNN architecture and trained, tested and validated with data from Utrecht UMC, Netherlands.

^b^Mean ECG age: arithmetic mean of ECG age_1_, ECG age_2_, ECG age_3_ used as a single predictor for each of the outcome (MI, Stroke, CVD mortality and Total mortality) model.

^c^All 3 ECG ages: model including ECG age_1_, ECG age_2_, and ECG age_3_ as three predictors.

## Discussion

In this study, we compared the estimated biological age of the heart referred to as ECG age using previously developed CNNs based on different architectures and trained on data collected from different populations. Specifically, we evaluated three models: (i) a CNN developed using architecture and data from the Mayo Clinic, (ii) a CNN built with the same architecture but trained on data from Utrecht UMC, and (iii) a CNN based on causalCNN architecture and trained on data from Utrecht UMC. We found that the ECG ages estimated from these three neural networks agreed well, irrespective of differences in neural network architecture and training data. The BA plots visually demonstrated fair pairwise agreement among the models. Moreover, numerical assessments of pairwise agreement using Pearson’s correlation coefficients, coefficient of variation and ICC indicated a good agreement. Additionally, the overall agreement assessed with ICC further confirmed a good agreement between CNN model outputs. In terms of predictive power, all the ECG age models showed similar C index values. The δ-age, a metric that indicates accelerated biological aging, was significantly associated with cardiovascular morbidities and mortalities across all models. Moreover, the 6-year predicted survival probabilities calculated using δ-age as the exposure variable showed very good agreement in both BA plots and ICC values. These findings suggest that despite variations in neural network architecture and training datasets, the models provide consistent estimates of ECG age that can be used for the prediction modeling of CVD.

Nevertheless, it is important to point out that the agreement between model outputs was not perfect, and some individuals had discrepant results in ECG age estimation. Both the architecture used to create a CNN and the population that was used to train the model (MN, USA or Utrecht, Netherlands) had an effect. Different sociodemographic characteristics and general health status of populations used for training the model may have played a role, as well as one architecture may be superior over another in providing more accurate predictions or causal clues to differences in aging biomarkers. Since there is not a golden standard for biological age estimation, we cannot state for sure that one model is superior over the other, or the reason for such discrepancies in ECG age estimation. More studies are needed to understand why some patients have discrepant ECG age estimates if produced by different CNNs, and what implications it can have for clinical predictions.

Although there are several AI models estimating biological ages of heart^3,13–15^, few studies have compared different AI models that estimate the ECG ages. A recent study compared^16^ four models, including the architecture used to obtain ECG age_1_^3^, but this work has considerable limitations. Only CNN architectures were compared by retraining them on a new population resulting in the CNNs differing from those in the original publication. Their performance was evaluated only with R-squared values and mean absolute errors without comparison of predictions of CVD events or mortality. Hence, to our knowledge, no studies have previously compared the pretrained models with validation on an external dataset. The significance and novelty of our study lie in the comparison of three previously proposed CNN models of biological aging based on ECG that differed by architecture and population used for training.

Building on these findings, we next consider their implications for clinical translation and generalizability. Previous research has demonstrated that AI models outperform traditional statistical methods, which makes them attractive for clinical use^9,17^. Since many AI models are developed by different research groups to estimate the biological age of the heart, it is essential to evaluate whether these models provide consistent results and can be used interchangeably in diverse settings. Consistency and reliability across different populations and settings are important for clinical and research applications of these models. By comparing three pre-trained CNNs for biological age, we offer insights into their performance on the external population. We demonstrate that they are suitable for age estimation in other populations without the need for repeated training, therefore facilitating their use in clinical applications aimed at CVD predictions. Our results have implications for medical imaging data sharing as they demonstrate that a model trained on hospital data is suitable for use in other regions and healthy populations. While medical image data sharing allows for saving considerable time, resources, and effort required for data collection^18^, validation of trained model on external datasets facilitates its further implementation in the clinical practice. Our findings also indicate that when multiple independently developed CNN models produce statistically agreeing ECG age estimates on a new external data, they are recognizing robust physiological patterns of cardiac aging rather than dataset-specific artifacts which doesn’t correlate to fundamental cardiac ageing feature, which in turn doesn’t generalize across data. In this study, all three CNNs, despite differing architectures and training cohorts, showed strong agreement, suggesting that ECG-derived age captures a genuine biological signal common across population. In practice, such multi-model agreement can be leveraged as a confidence indicator for ECG age predictions. Since there is no gold standard for biological heart age, obtaining an ages with good agreement from all three CNNs provides an internal consistency check, implying that the prediction is likely reliable and robust. This consensus acts as a surrogate confidence score, analogous to how concordant assessments by different physicians increase diagnostic confidence.

These implications should be interpreted in light of the study’s strengths and limitations. We used several indices to assess the agreement of the ECG ages estimated by CNN models in the external dataset (the Tromsø Study). Not only did we evaluate the predictive power of ECG age on health outcomes, but we also compared the associations of accelerated aging, as determined by the δ-age, with these outcomes. The Tromsø Study (2015–2021) used for our comparison study has advantages of prospective study design, high participation, minimal missing questionnaire data, and rigorous medical test procedures.

The limitation of this study is the comparison between CNNs only using the Tromsø Study data. The agreement between biological aging biomarkers estimated by the three CNNs may be specific to the Tromsø Study participants, therefore limiting the generalizability of the study results. Additionally, our comparisons were restricted to three CNNs each trained on rather homogenous populations of either MN, USA or Utrecht, Netherlands, which consists predominantly of individuals of white race. This lack of diversity may bias or limit the models’ predictive power in populations with different ethnicities or sociodemographic profiles.

In conclusion, we compared ECG ages estimated from three different CNNs and found that they agree well despite variations in model architecture and training data. All models demonstrated comparable accuracy and good predictive power for cardiovascular disease and mortality outcomes. The δ-ages derived from these ECG ages were significantly associated with these health outcomes, reinforcing the utility of ECG-based biological age estimation. Moreover, the close agreement among the three models implies that each network is identifying the same underlying biological signal of cardiac aging in the ECG rather than model-specific artifacts.

Pretrained models can save substantial computation time and resources, making them more accessible for widespread clinical use. Hence by assessing the agreement between different neural networks trained on diverse datasets, we demonstrate their generalizability across different populations. In practice, having multiple models having strong agreement between their predictions can serve as a surrogate confidence measure when no gold standard for heart age exists. Agreement among independent models effectively provides an extra layer of validation for the predicted age. More such cross comparisons are needed to test whether the relatively comparable performance of the CNNs examined here is consistent across other populations. In our study, we underscore the potential for these models to provide consistent and reliable estimates in various clinical and research settings, ultimately advancing personalized health assessments and good clinical predictions.

## Methods

### Study design and sample

The Tromsø Study is a population-based study conducted since 1974 in the municipality of Tromsø, Norway^19^. The present analysis includes data collected in Tromsø7 (2015–2016)^20^. All residents of Tromsø municipality aged 40 years or older were invited to Tromsø7, and 21,083 (65%) participated. The Tromsø7 Study consisted of two visits; the first visit was attended by all participants, included questionnaires and clinical examination with collection of basic measurements such as height, weight, blood pressure and blood samples. The second visit was attended by a subsample of 8346 participants and out of them 7778 participants were examined with 12-lead ECG. Participants with prevalent myocardial infarction (*n* = 377) and stroke (*n* = 200) were excluded along with participants who had incomplete covariate data (*n* = 124). A final sample of 7108 participants were included in the study.

### Ethical approval

The Tromsø Study complies with the Declaration of Helsinki and has been approved by the Regional Committee for Medical and Health Research Ethics (REK), the Data Inspectorate, and the Norwegian Directorate of Health. All participants provided written informed consent. The current study was approved by REK Nord (reference 68185) and a data management plan was evaluated by the Norwegian Centre for Research Data.

### Measurements

#### Exposure

ECG age (ECG age_1_, ECG age_2_ and ECG age_3_) was estimated by applying three different CNNs, varying in network architecture, training data or both, to 12-lead ECGs obtained from the 7778 participants of the second visit of Tromsø7. Apart from ECG age, several other terms have been employed in the literature to refer to the same concept, including “heart age”, “cardiac age”, “vascular age”,” ECG Heart Age”, “AI-ECG–predicted age”, “AI ECG-heart age”, “CNN-predicted age”. As an additional metric for comparison, δ-ages (δ-age_1_, δ-age_2_ and δ-age_3_) were calculated by subtracting chronological age from ECG ages mentioned above. Similar to the ECG age, for the δ-age, terms like “heart delta age”, “ECG-age gap”, “age gap” and “delta age” are also commonly used.

The details on CNN architecture, training and validation are described in Table 5. Briefly, the CNN1 for ECG age estimation was developed by our team using data collected from Mayo Clinic digital data vault of individuals over 18 years of age. Standard 10 s 12-lead samples of ECGs were used as an input, with the output being the AI-predicted ECG age as a continuous variable. This neural network consists of stacked blocks of convolutional, max pooling, and batch normalization, with a non-linear activation function following each block^3,21^ and achieved a mean absolute error (MAE) of 6.9 years and an R-squared of 0.7 on validation data. We used CNN1 to predict the ECG age_1_ for Tromsø7 participants.

**Table 5:** Characteristics of Convolutional Neural Networks used to estimate ECG age for population of Tromsø7

	CNN1	CNN2	CNN3
Implementation	Python using Keras with a Tensorflow (Google, Mountain View, CA, USA) backend	PyTorch	PyTorch
Network architecture	After the first group of 8 layers extracted temporal features, another spatial block was used to fuse data from all leads, and then the extracted features were used in a fully connected network (2 layers). The output layer had linear activation function, selected hyperparameters were learning rate of 3e–4 and batch size of 64, a mean standard error loss was used as a loss function.	After the first group of 8 layers extracted temporal features, another spatial block was used to fuse data from all leads, and then the extracted features were used in a fully connected network (2 layers). The output layer had linear activation function, selected hyperparameters were learning rate of 0.001 and batch size of 64, a mean standard error loss was used as a loss function.	CausalCNN: seven dilated causal convolutions blocks were constructed. Each causal convolution block consists of a combination of causal convolutions, weight normalizations, leaky ReLUs and residual connections. The dilation in the convolutional layer is exponentially doubled each block from 1 to 64. For the first 6 blocks, the number of output channels is kept constant at 108 and the final 7th block outputs 216 channels. All convolutional layers, except for the residual connections, used a kernel size of 3 and a value of 0.01 was used for the negative slope parameter of the leaky ReLU activation functions.
Population used for training, testing, and validation	Mayo Clinic	University Medical Centre Utrecht (UMCU)	University Medical Centre Utrecht (UMCU)
Population source (hospital patients vs community)	Patients	Patients	Patients
*N*	Training (*N* = 399,750), internal validation (*N* = 99,977), and testing (*N* = 275,056)	Training (*N* = 1,030,958) and validation (*N* = 116,815)	Training (*N* = 1,030,958) and validation (*N* = 116,815)
Age range health	>18 years old, mean = 58.6 (SD = 16.2)	>18 years old, mean = 59 (SD = 16)	>18 years old, mean = 59 (SD = 16)
MAE	6.9 years	7.2 years	7.0 years
ECG acquisition	Sampling rate of 500 Hz using a GE-Marquette ECG machine (Marquette, WI) and stored using the MUSE data management system.	Sampled at both 250 and 500 Hz and linear interpolation was used to resample all ECGs to 500 Hz. Acquired with General Electric MAC ECG device and retrieved from the MUSE ECG system in digital format.	Sampled at both 250 and 500 Hz and linear interpolation was used to resample all ECGs to 500 Hz. Acquired with General Electric MAC ECG device and retrieved from the MUSE ECG system in digital format.
Code availability	Patented	Not published	Available on https://github.com/UMCUtrecht-ECGxAI/ecgxai.

The CNN2 for ECG age estimation was developed using the same neural network architecture as in CNN1^3^ but utilized data from the University Medical Centre Utrecht (UMCU) for training and validation. The dataset included all patients over the age of 18 years and ECGs of insufficient quality were excluded. CNN2 achieved an MAE of 7.2 years and an R-squared of 0.7. This newly trained CNN2 was used to estimate ECG age_2_ for Tromsø7 participants.

The CNN3 model for ECG age estimation was developed using CausalCNN model architecture, the same data from UMCU as in CNN2. The CausalCNN architecture has been described in detail before^22–24^. This deep CNN featured seven dilated causal convolutional blocks with exponentially increasing dilation factors. CNN3 achieved an MAE of 7.0 years and an R-squared of 0.7 and was used to estimate ECG age_3_ for Tromsø7 participants.

### Baseline covariates

Baseline covariates were collected during the first visit of Tromsø7. Daily smoking status (yes/no) was collected from self-reported questionnaires. Systolic blood pressure was measured on the right arm three times at one-minute intervals after 2 minutes of seated rest with a Dinamap ProCare 300 (GE Healthcare, Norway), and the mean of the two last readings was used in the analyses. Non-fasting venous blood samples were collected with the participant sitting and a brief venous stasis applied to the upper arm was released before venipuncture. The analysis was done on the fresh blood samples within 48 h at the Department of Laboratory Medicine, University Hospital of North Norway, Tromsø. Serum total cholesterol concentrations were analyzed by CHOD-PAP enzymatic colorimetric methods with Roche Diagnostics, Mannheim, Germany); high-density lipoprotein (HDL)-cholesterol was measured after separating apoB-containing lipoproteins by using heparin and manganese chloride; non-HDL cholesterol was calculated by subtracting HDL-cholesterol from serum total cholesterol.

### Outcome

Myocardial infarction (MI) and stroke, both prevalent and incident, were recorded from multiple sources. Events up to 2014 came from the Tromsø Study’s local cardiovascular disease register^25,26^, while those from 2015 to 2021 were obtained from national registries of myocardial infarction and stroke. The local registry was formed by linkage to diagnoses registered at the University Hospital of North Norway and the National Causes of Death Registry. All Norwegian hospitals are mandated to register patients hospitalized with acute MIs to the Norwegian Myocardial Infarction Register and acute strokes to the Norwegian Stroke Register since 2012. MI inclusion criteria were patients with an I21 or I22 diagnosis hospitalized within 28 days after symptom onset; for stroke, those admitted with I61, I63, or I64. Stroke Register did not include strokes following a traumatic head injury, stroke related to intracranial tumors, or ischemic stroke following a subarachnoid hemorrhage. The National Cause of Death Registry from the Norwegian Institute of Public Health supplied data on fatal CVD, including out-of-hospital, and all-cause mortality cases. Deceased individuals aged 40+ with CVD (ICD-10 codes I00–I82) listed as the primary cause on their death certificate were included for CVD mortality model and all deaths covering 2015–2021 were included for all-cause mortality analysis. The diagnoses recorded in the national registers overlapped with the local register for 2013 to 2014 and have been verified to be in agreement and complete when compared with the validated diagnoses in the local register^27,28^.

### Statistical analysis

Baseline characteristics were presented as means and standard deviations (SD) for continuous variables and numbers and percentages for categorical variables.

### Accuracy of ECG age estimations based on different CNNs

Mean absolute error (MAE), defined as the average absolute difference between ECG ages estimated from the different CNNs and chronological age were calculated and contrasted to the original MAE reported for each respective CNN.

### Agreement between ECG age estimations based on different CNNs

Pearson’s correlation coefficients were computed to assess the correlation between the ECG ages and chronological age, and between ECG ages. We presented Bland–Altman (BA) plots to visually assess agreement between the three ECG ages. Limits of agreement in a BA plot represent a range where 95% of the differences between a pair of ECG ages are expected to be. To support the use of BA plots, we visually inspected the distributions of the pairwise differences via histograms and assessed whether the variance of the differences was approximately constant across the range of values using the BA plots itself. We estimated pairwise and overall intraclass correlation coefficients (ICC(3,1)) as numeric measures of agreement as defined by McGraw and Wong^29^. ICC(3,1) is the two way mixed effect model type of ICC that evaluates the reliability of a measurement made by each rater. Additionally, we also assessed the relative variability between ECG ages using coefficient of variation (CV). We also computed Lin’s concordance correlation coefficient (CCC)^30^ and the calibration slope for each pair of ECG ages.

### Prediction of CVD outcomes using δ-age based on different models

We used Cox proportional hazard models to estimate six-year survival probabilities for each outcome MI, stroke, CVD mortality and total mortality, separately for each exposure variable δ-age_1_, δ-age_2_, and δ-age_3_. Follow-up time ranged from the day of study entry in 2015–16 to the date of first event, participant censoring due to emigration from Norway, death, or the end of follow-up on December 31, 2021, whichever came first. This was done in models including each δ-age along with chronological age and sex. The survival probabilities were compared for their agreement and reliability using the BA plot and ICC(3,1). Cox models were also used to estimate hazard ratios (HRs) for each outcome per SD increase in δ-age, in models adjusted for chronological age and sex and then additionally adjusted for daily smoking, non-HDL cholesterol and systolic blood pressure.

Finally, we used 8-fold cross-validation^31^ to assess and compare the predictive accuracy and reliability of the Cox models in our study. For each outcome, participants were randomly assigned to one of eight approximately equal-sized (*N* = 877 or 878) folds. In each cycle, a Cox proportional hazards model was fitted on seven folds and evaluated on the remaining fold. Harrell’s concordance index (C-index) was computed from the model’s linear predictor. All model specifications (each ECG-age predictor or combination, alongside chronological age) used the same fold assignments for a given outcome. For each of the four outcomes, we fit separate Cox models multiple times, replacing the ECG-age predictor in turn with (i) ECG age_1_, (ii) ECG age_2_, (iii) ECG age_3_, (iv) the average of the three ECG ages, and (v) a combination including all three ECG ages separately as predictors; all models additionally included chronological age. The C-index was computed for each model in each fold, and model performance was summarized by the mean C-index across the eight folds. We obtained 95% confidence intervals for the mean C-index using 1000 non-parametric bootstrap samples, repeating the full 8-fold cross-validation procedure within each bootstrap replicate. The inclusion of chronological age is to reduce the bias in estimation of biological age^32,33^. Upon the completion of the cross-validation cycles, the performance of the model was assessed by the average value of C-index across all 8 folds.

## Supplementary information

41514_2026_344_MOESM1_ESM

41514_2026_344_MOESM2_ESM

41514_2026_344_MOESM3_ESM

## Data Availability

Data may be obtained from a third party and are not publicly available. The data supporting the findings in this study are available through an application directed to The Tromsø Study by following the steps presented on their webpage. https://uit.no/research/tromsostudy.

## Code availability

The underlying code for this study is available in the GitHub repository (https://github.com/Arya200196/PhD_ECG-age-comparison).
