# Supplementary material for: Electrocardiogram derived heart age models agreement, accuracy and predictive ability in the Tromsø study
Source: NPJ Aging. 2026 Mar 20;12(1):72. doi: 10.1038/s41514-026-00344-2 (PMC13223277; doi:10.1038/s41514-026-00344-2)
Supplement: Supplementary file 1 — 41514_2026_344_MOESM1_ESM [file 41514_2026_344_MOESM1_ESM.docx]

**Supplementary Materials**


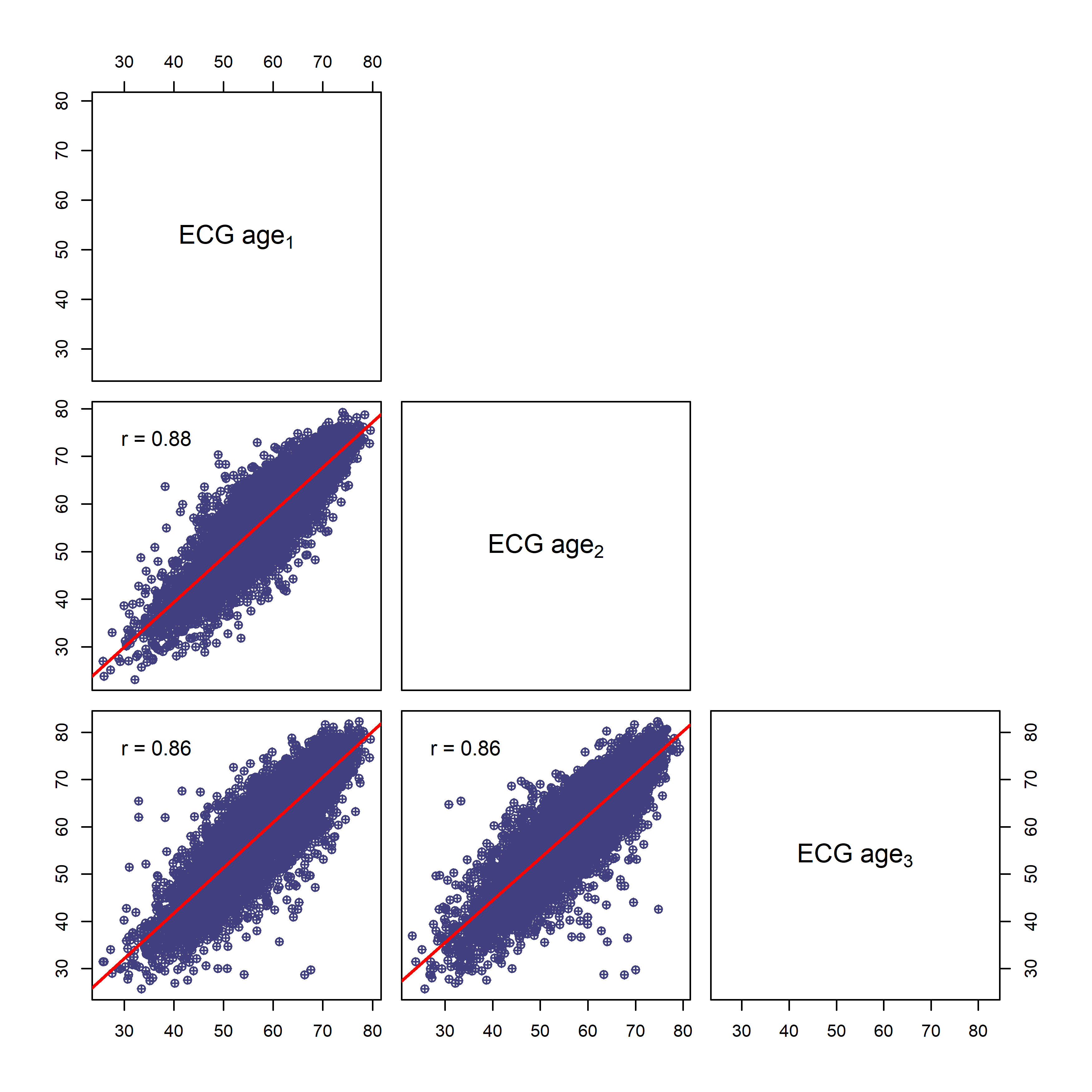
**Supplementary Figure 1**: Scatter plot matrix showing the pairwise correlations between estimated ECG ages (ECG age_1_, ECG age_2_, and ECG age_3_). (i) ECG age_1_: estimated by CNN1 model trained, tested and validated with data from Mayo Clinic, MN, USA; (ii) ECG age_2_: estimated by CNN2 model based on Mayo Clinic’s model architecture and trained, tested and validated with data from Utrecht UMC, Netherlands; (iii) ECG age_3_ estimated by CNN3 model based on CausalCNN architecture and trained, tested and validated with data from Utrecht UMC, Netherlands. Each subplot represents the relationship between two ECG age measurements (ECG age_1_ v/s ECG age_2_, ECG´age_2_ v/s ECG age_3_ and ECG age_1_ v/s ECG age_3_), with a red line indicating the trend. Pearson correlation coefficients are provided at the top left for each pair.

**Supplementary Figure 2:** The BA plots illustrate the agreement (with 95% limits of agreement) between the 6-year survival probability (in %) of MI from the survival models with each of δ-age_1_, δ-age_2_ and δ-age_3_, variable as exposure. δ-age_1_ is calculated from the ECG age_1_, estimated by CNN1 model with data from Mayo Clinic, MN, USA, δ-age_2_ from ECG age_2_, estimated by CNN2 model based on Mayo Clinic’s model architecture and trained, tested and validated with data from Utrecht UMC, Netherlands, δ-age_3_ from ECG age_3_, estimated by CNN3 model based on CausalCNN architecture and trained, tested and validated with data from Utrecht UMC, Netherlands. Each panel from left to right represents a pair-wise comparison: (A) 6-year survival probability (%) for the exposure δ-age_1_ v/s δ-age_2_ (B) 6-year survival probability (%) for the exposure variable δ-age_2_ v/s δ-age_3_, and (C) 6-year survival probability (%) for the exposure variable δ-age_1_ v/s δ-age_3_


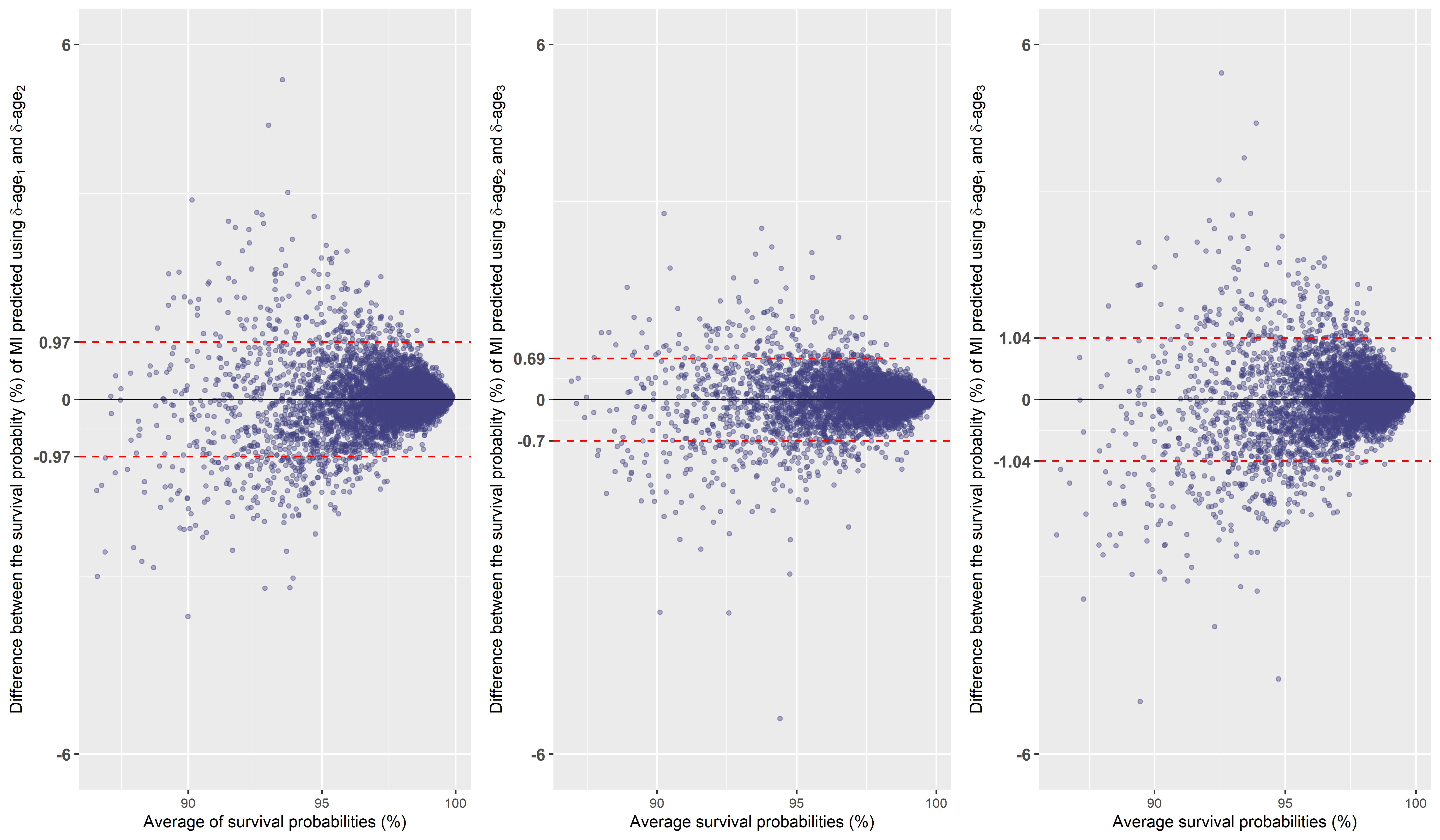
 **Supplementary Figure 3:** The BA plots illustrate the agreement (with 95% limits of agreement) between the 6-year survival probability (in %) of stroke from the survival models with each of δ-age_1_, δ-age_2_ and δ-age_3_, variable as exposure. δ-age_1_ is calculated from the ECG age_1_, estimated by CNN1 model with data from Mayo Clinic, MN, USA, δ-age_2_ from ECG age_2_, estimated by CNN2 model based on Mayo Clinic’s model architecture and trained, tested and validated with data from Utrecht UMC, Netherlands, δ-age_3_ from ECG age_3_, estimated by CNN3 model based on CausalCNN architecture and trained, tested and validated with data from Utrecht UMC, Netherlands. Each panel from left to right represents a pair-wise comparison: (A) 6-year survival probability (%) for the exposure δ-age_1_ v/s δ-age_2_ (B) 6-year survival probability (%) for the exposure variable δ-age_2_ v/s δ-age_3_, and (C) 6-year survival probability (%) for the exposure variable δ-age_1_ v/s δ-age_3._


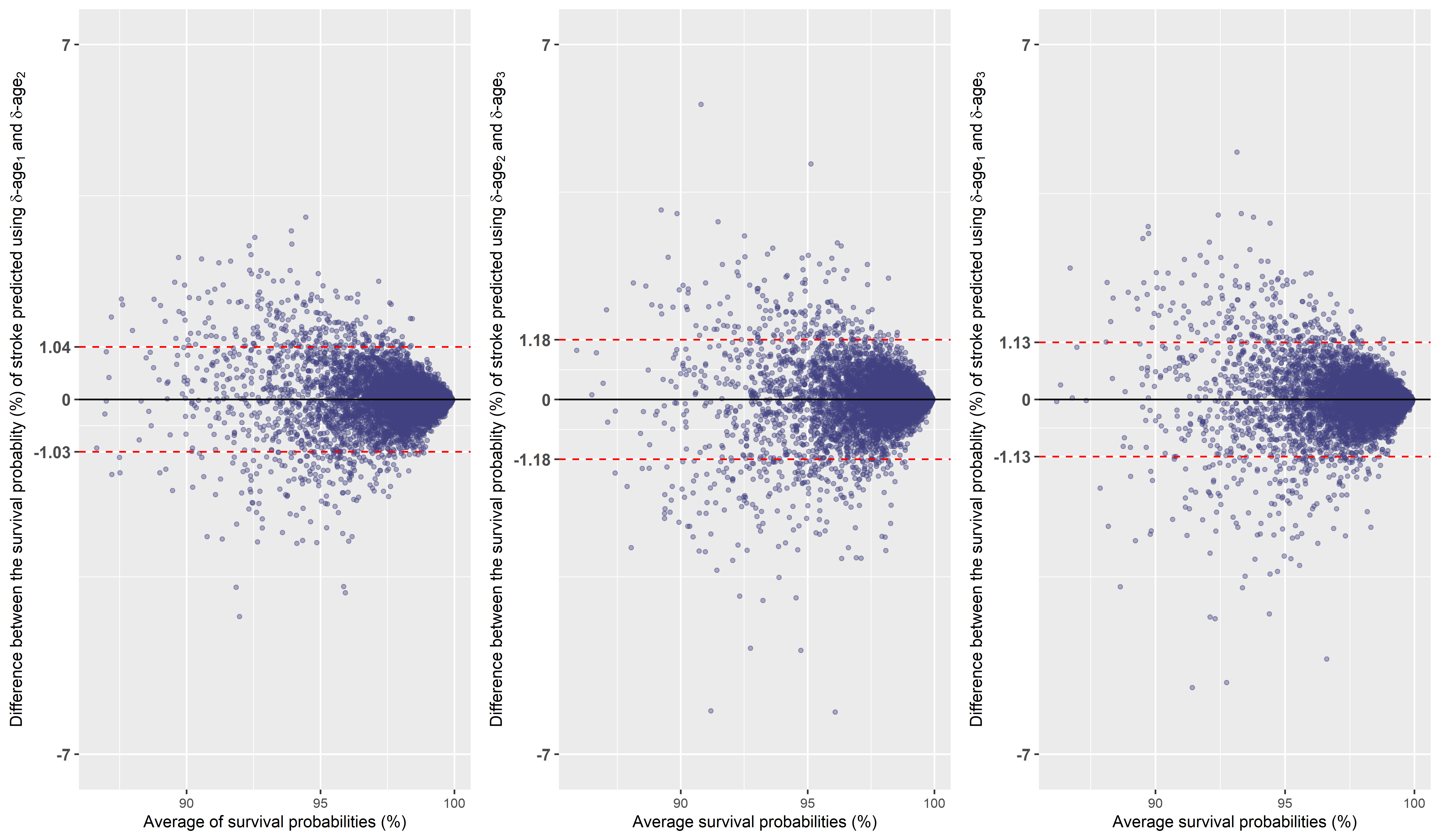


**Supplementary Figure 4:** The BA plots illustrate the agreement (with 95% limits of agreement) between the 6-year survival probability (in %) of CVD mortality from the survival models with each of δ-age_1_, δ-age_2_ and δ-age_3_, variable as exposure. δ-age_1_ is calculated from the ECG age_1_, estimated by CNN1 model with data from Mayo Clinic, MN, USA, δ-age_2_ from ECG age_2_, estimated by CNN2 model based on Mayo Clinic’s model architecture and trained, tested and validated with data from Utrecht UMC, Netherlands, δ-age_3_ from ECG age_3_, estimated by CNN3 model based on CausalCNN architecture and trained, tested and validated with data from Utrecht UMC, Netherlands. Each panel from left to right represents a pair-wise comparison: (A) 6-year survival probability (%) for the exposure δ-age_1_ v/s δ-age_2_ (B) 6-year survival probability (%) for the exposure variable δ-age_2_ v/s δ-age_3_, and (C) 6-year survival probability (%) for the exposure variable δ-age_1_ v/s δ-age_3_


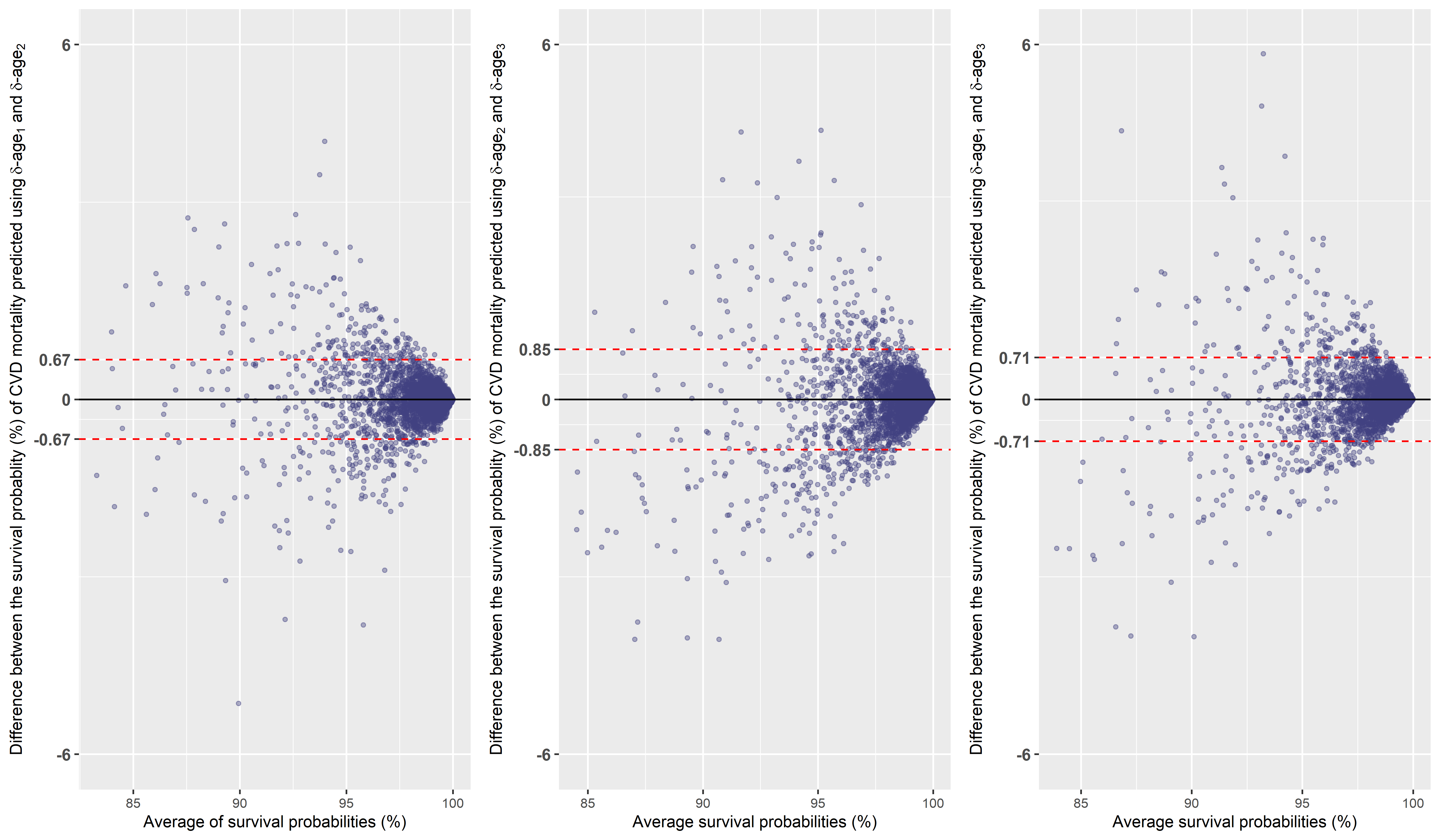


**Supplementary Figure 5:** The BA plots illustrate the agreement (with 95% limits of agreement) between the 6-year survival probability (in %) of total mortality from the survival models with each of δ-age_1_, δ-age_2_ and δ-age_3_, variable as exposure. δ-age_1_ is calculated from the ECG age_1_, estimated by CNN1 model with data from Mayo Clinic, MN, USA, δ-age_2_ from ECG age_2_, estimated by CNN2 model based on Mayo Clinic’s model architecture and trained, tested and validated with data from Utrecht UMC, Netherlands, δ-age_3_ from ECG age_3_, estimated by CNN3 model based on CausalCNN architecture and trained, tested and validated with data from Utrecht UMC, Netherlands. Each panel from left to right represents a pair-wise comparison: (A) 6-year survival probability (%) for the exposure δ-age_1_ v/s δ-age_2_ (B) 6-year survival probability (%) for the exposure variable δ-age_2_ v/s δ-age_3_, and (C) 6-year survival probability (%) for the exposure variable δ-age_1_ v/s δ-age_3_

_
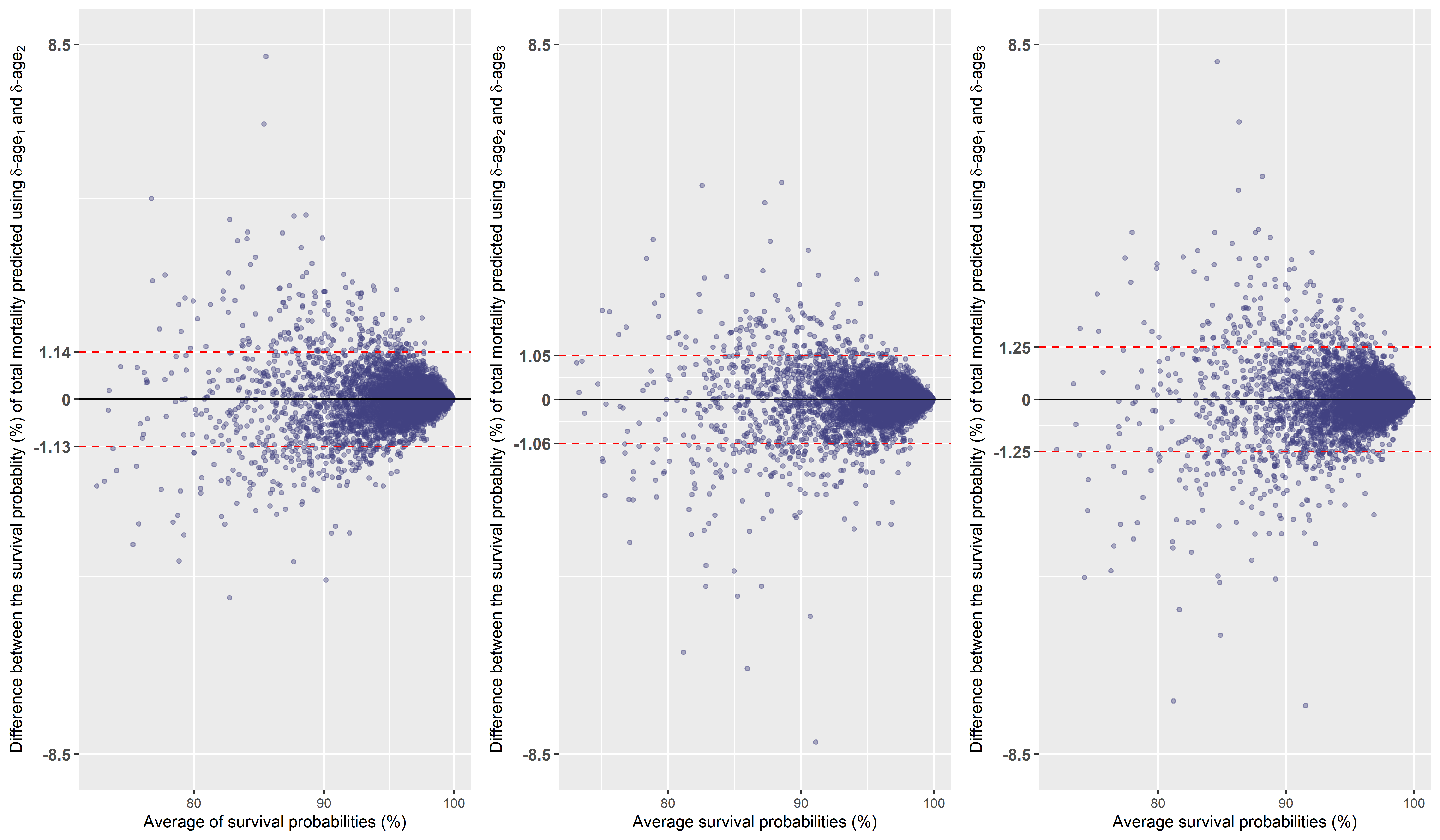
_

**Supplementary** **Table 1**: Pairwise agreement between convolutional neural network–derived ECG-age estimates: Lin’s concordance correlation coefficients (CCC) and bidirectional calibration slopes.

| **Pair of ECG-age models** | **Lin’s CCC** | **Calibration slope: second on first* (95% CI)** | **Calibration slope: first on second† (95% CI)** |
| --- | --- | --- | --- |
| ECG age1 vs ECG age2 | 0.86 | 0.81 (0.80, 0.82) | 0.94 (0.93, 0.96) |
| ECG age1 vs ECG age3 | 0.85 | 0.77 (0.76, 0.78) | 0.96 (0.95, 0.97) |
| ECG age2 vs ECG age3 | 0.83 | 0.83 (0.82, 0.84) | 0.89 (0.88, 0.91) |

*Calibration slopes estimated from linear regression of the second model’s ECG-age prediction on the first model’s ECG-age prediction (e.g. ECG age2 on ECG age1).
†Calibration slopes estimated from linear regression of the first model’s ECG-age prediction on the second model’s ECG-age prediction (e.g. ECG age1 on ECG age2). CCC, Lin’s concordance correlation coefficient.
